# Parasite genotypes and host immune mediators in primary and recurrent episodes of vivax Malaria in Colombia

**DOI:** 10.17843/rpmesp.2025.424.14865

**Published:** 2025-12-13

**Authors:** Yesica Yamile Duque-Isaza, Jaime Carmona-Fonseca, Eliana María Arango-Flórez

**Affiliations:** 1 Salud y Comunidad-César Uribe Piedrahíta Group, Faculty of Medicine, Universidad de Antioquia, Medellín, Colombia. Universidad de Antioquia Salud y Comunidad-César Uribe Piedrahíta Group Facultad de Medicina Universidad de Antioquia Medellín Colombia; 2 GEINCRO, Faculty of Health Sciences, Fundación Universitaria San Martín, Sabaneta, Colombia. Universidad San Martín GEINCRO Faculty of Health Sciences Fundación Universitaria San Martín Sabaneta Colombia

**Keywords:** Malaria, Plasmodium vivax, Recurrence, Pregnancy, Genotypes, Cytokines

## Abstract

**Objectives.:**

To identify *Plasmodium vivax* genotypes in the pregnant and non-pregnant population and explore their association with immune mediators.

**Materials and methods.:**

Two cohorts of patients with uncomplicated vivax malaria were followed for 120 days in Puerto Libertador and Tierralta, Cordoba, Colombia: 41 pregnant women and 46 non-pregnant individuals, all treated with standard treatment. Parasite genotypes (microsatellites Pv3.27, Pv3.502, and Pv1.501) and the expression of host immune mediators (IL-13, IL-10, TNF-alpha, IFN-gamma, IL-8, TGF-beta, Fox-P3, PD-L1) were compared between primary and recurrent infections.

**Results.:**

The frequency of recurrences was higher in pregnant women (41.6%) than in non-pregnant individuals (8.7%). Parasite genetic diversity was higher in pregnant women, although without exclusive alleles. Recurrences were genetically homologous (same alleles) in only 23% and 33% of cases in pregnant and non-pregnant individuals, respectively. Regarding immune mediators, only Fox-P3 expression varied significantly in pregnant women, with higher expression in the primary episode than in the recurrent one.

**Conclusions.:**

Pregnant women are exposed to a high frequency of vivax malaria recurrences, with high parasite genetic variation, which may affect the development of effective protective immunity and favor adverse obstetric outcomes, both maternal and neonatal. These findings raise the need to strengthen and optimize prevention and treatment actions for *vivax* malaria during pregnancy.

## INTRODUCTION

Infection by *Plasmodium vivax* during pregnancy is associated with disease in the woman (gestational malaria), placental damage (placental malaria) ^1^, and adverse maternal and perinatal outcomes ^2^^,^^3^. It is estimated that, each year, more than 90 million pregnancies worldwide are at risk of *P. vivax* infection ^4^. In particular, in the American continent, this species is responsible for more than 80% of malaria cases ^5^, with reports from northwestern Colombia documenting a frequency of gestational malaria of 35.8% and placental malaria between 20.9% and 27.7% ^6^^,^^7^. Studies that contribute to the knowledge of the genetics and pathogenicity of the parasite, as well as the host immune response, are indispensable for understanding the pathophysiological processes of this species. In general, knowledge about *P. vivax* is much less advanced than that of *P. falciparum*. However, available reports leave no doubt about the pathogenic role of *P. vivax* during gestation nor about the morbid effects it produces in pregnant women and their children ^1^^-^^3^^,^^8^^,^^9^.

All *Plasmodium* species can cause malaria recurrences, understood as the reappearance of parasitemia, with or without associated symptoms, after having received specific antimalarial treatment ^10^^,^^11^. These recurrences can be of three types: A) recrudescence, caused by the incomplete elimination of erythrocytic stages (therapeutic failure). It usually appears within 28 days of initiating treatment and can be caused by any species of *Plasmodium*^10^^,^^12^. B) Reinfection, generated by the inoculation of new sporozoites by the vector. It occurs after 28 days of initiating treatment in patients with complete cure of the initial episode, and can be caused by any species ^10^^,^^12^^,^^13^. C) Relapse, caused by the activation of hypnozoites (latent hepatic stages) and appears after 28 days of initiating treatment for *P. vivax* or *P. ovale* malaria, in patients who had a complete resolution of the primary episode ^10^^,^^12^^-^^14^.

Other suggested sources of relapses are erythrocytic stages sequestered in the spleen and bone marrow ^15^, sporozoites that survive in the skin, and merozoites present in the lymphatic system ^16^. In patients coming from non-endemic areas, the differentiation between recrudescence, reinfection, and relapse can be performed through clinical and parasitological follow-up. However, in residents of endemic areas, this differentiation is limited, since currently there are no accessible tools available that allow distinguishing reliably between relapses and reinfections ^17^.

Primaquine (8-aminoquinoline) as a hypnozoiticide, in combination with chloroquine (4-aminoquinoline), which acts as a blood schizonticide, constitutes the treatment indicated to cure vivax malaria and prevent relapses in Colombia and in most endemic areas. However, primaquine is contraindicated during pregnancy and lactation due to the risk of dose-dependent hemolysis in fetuses and newborns with undiagnosed glucose-6-phosphate dehydrogenase deficiency ^18^^,^^19^.

The quantity of recurrences by *P. vivax* could be influenced by immunity acquired during the primary episode and by the immunological history of previous episodes ^20^. Patients acquire natural immunity after primary infection, and experimental studies have demonstrated that immune protection is clone-specific ^21^. However, available information on the immune response in malaria recurrences is scarce ^22^^,^^23^. In Thailand, it was observed that both proinflammatory and anti-inflammatory cytokines presented higher levels in patients with *P. vivax* than in healthy individuals. Furthermore, in patients with homologous or heterologous relapses, IL-1β levels were higher during primary infection, while IL-6 and IL-10 levels were higher in heterologous relapses than in homologous ones ^21^.

The genetic diversity of *P. vivax* is much greater than that of *P. falciparum*^24^^,^^25^. Genotypically, malaria recurrences can be homologous or heterologous: in homologous ones, the parasite genotypes of the primary episode and the recurrence are exactly the same ^26^^,^^27^, while in heterologous ones, different parasite genotypes are found in the primary episode and in the recurrence ^27^. In Colombia, few studies have compared *P. vivax* genotypes between primary infection and recurrence ^28^^,^^29^, and none have been conducted in the pregnant population. The human host immune response to plasmodial infection is very complex, and when it occurs during gestation, this complexity is even greater due to the hormonal and immunological changes that occur during pregnancy, where it is necessary to maintain a balance between the proinflammatory and anti-inflammatory immune response ^30^.

This study aimed to compare *P. vivax* genotypes between the primary episode and the recurrence in the pregnant and non-pregnant population, as well as to explore the association between parasite genotypes present in those malaria episodes with the expression of proinflammatory and anti-inflammatory immune mediators in both groups.

**KEY MESSAGES**

Motivation for the study. Vivax malaria constitutes a relevant challenge in public health in Colombia, especially in pregnant women, who are more prone to recurrences and complications. However, the genetic diversity of the parasite in primary and recurrent infections, as well as its relationship with the host immune response, has been scarcely studied.

Main findings. Pregnant women presented a higher frequency of recurrences (41%) and a greater genetic diversity of the parasite compared to non-pregnant individuals. Likewise, a higher expression of Fox-P3 was observed during the primary infection of pregnant women, which may modulate protective immunity.

Public health implications. Strengthening the prevention and control of *P. vivax* malaria in pregnancy is crucial to decrease recurrences and improve maternal and neonatal outcomes.

## MATERIALS AND METHODS

### Study site and population

This research was executed in Puerto Libertador (7°53′17″N 75°40′25″W) and Tierralta (8°10′22″N 76°03′34″W), in the department of Cordoba, Colombia. Both belong to the second malaria transmission focus in Colombia (Uraba-Bajo Cauca-Sinu-San Jorge), where *P. falciparum* and *P. vivax* co-circulate, although *P. vivax* predominates, with 65% of cases ^31^^,^^32^. Approximately 51% of vivax malaria cases reported in Colombia come from this region. Between the years 2000 and 2023, 674,294 accumulated cases were registered, with an annual parasite index for *P. vivax* (number of cases per 1000 inhabitants) of 13.9 ^32^.

### Study design

A descriptive, prospective, and longitudinal study was carried out, with two cohorts: 41 pregnant women and 46 non-pregnant men and women, all volunteers with a diagnosis of uncomplicated malaria caused exclusively by *P. vivax*. The infection was detected by thick blood smear (TBS) and confirmed by quantitative real-time PCR (qPCR). The inclusion of participants was progressive between 2016 and 2019, both at the malaria diagnostic post of the local hospital and in prenatal consultation.

### Selection criteria

*Inclusion criteria*

Pregnant women of any gestational age were invited to participate, as well as non-pregnant men and women, who presented single infection by *P. vivax* confirmed by TBS and qPCR, who were classified as uncomplicated malaria cases, and who voluntarily consulted at the local hospital between 2016-2019. All participants consented to their voluntary participation by signing the informed consent, and in cases of minors, informed assent was also signed.

*Exclusion criteria*

Pregnant women with a history of comorbidities compatible with TORCHS syndrome, as registered in the clinical history, were excluded, as well as patients (pregnant or non-pregnant) who had received any antimalarial other than the standard treatment prescribed at the local hospital. Those who withdrew consent, developed any malaria complication during follow-up, or reported pregnancy during the course of the study within the non-pregnant cohort were also excluded.

### Antimalarial treatment

The treatment supplied to the volunteers of this study was that recommended by the World Health Organization (WHO) and the health authorities of Colombia for uncomplicated *P. vivax* malaria. In non-pregnant patients, the combination of chloroquine for three days (total dose of 25 mg/kg) plus primaquine for 14 days (daily dose of 0.25 mg/kg) was administered. In pregnant women, only chloroquine was prescribed (total dose of 25 mg/kg divided over three days), due to the contraindication of primaquine during pregnancy ^18^^,^^33^. The treatment was dispensed by the staff of the malaria diagnostic post of the local hospital, and its administration was unsupervised.

### Sample collection and follow-up

On the day of admission to the study (day 1), before the administration of the first dose of antimalarial treatment, venopuncture was performed on each volunteer to obtain a peripheral blood sample, to prepare: a) two TBS slides for microscopic diagnosis of malaria; b) three circles of Whatman® No. 3 filter paper for DNA extraction for confirmation of single *P. vivax* infection by qPCR and for genotyping of parasites with microsatellites; c) two vials, each with 100 μL of the leukocyte pellet mixed with 900 μL of TRIzol (Invitrogen), preserved in liquid nitrogen until use in RNA extraction for the quantification of immune mediator expression.

Follow-up for the detection of recurrences was performed on days 28, 60, 90, and 120 after the start of antimalarial treatment. At each follow-up, malaria symptomatology was evaluated and venopuncture was performed to collect the same samples obtained on the day of admission. Unscheduled visits by volunteers between days 2 and 120 were also considered for the detection of recurrences; that is, if any volunteer presented a positive TBS or malaria symptomatology on days other than 28, 60, 90, or 120, it was also considered a malaria recurrence and, on that day, the same blood samples as on the day of admission were collected.

### Diagnosis of plasmodial infection

TBS slides were stained with Field stain and read by an experienced microscopist technician from the local hospital, following WHO recommendations ^34^. For parasitemia calculation, a parasite count was performed in a total of 200 leukocytes, using the constant of 8000 leukocytes/μL.

Confirmation of single *P. vivax* infection was done by qPCR, using the protocol and primers and probes for the 18S gene, which were designed by researchers from Edmonton, Canada ^35^. DNA extraction from filter paper was done using the Saponin- Chelex method previously described by other researchers ^36^.

### Parasite genotyping

Amplification of microsatellites Pv3.27, Pv3.502, and Pv1.501 was done by semi-nested PCR according to Koepfli *et al*. ^37^, with some modifications: 1X PCR buffer (Qiagen), 3 mM (Pv3.27) or 3.5 mM (Pv3.502, Pv1.501) of MgCl2 (Qiagen), 200 μM of each deoxynucleotide triphosphate (dNTP) (Takara Bio), 0.25 μM of each primer, and 0.067 IU/ μL of recombinant Taq DNA polymerase (Fermentas). To confirm adequate amplification, 2 μL of each product were run on 2% agarose gel electrophoresis, stained with GelRed™ (Biotium), and bands were visualized on a UV transilluminator.

Analysis of the size of amplified products was performed by capillary electrophoresis on a 3500 genetic analyzer (Hitachi Applied Biosystems), with the internal standard GeneScan 600 LIZ (Applied Biosystems) and factory conditions for microsatellites. PCR products were diluted in Milli-Q water, 1:10 (Pv3.502) or 1:20 (Pv1.501 and Pv3.27). Electrophoresis data were analyzed with GeneMapper ID-X Software v1.5 (Applied Biosystems) and electropherograms were examined visually. Peaks above 300 relative fluorescence units (RFU) were considered true amplifications. Alleles were grouped manually according to their size, as follows: groups of 4 bp for Pv3.27; 7 bp for Pv1.501, and 8 bp for Pv3.502 ^38^. Polyclonal infection was established when more than one peak was observed at a locus and the height of the minor peak was greater than 33% of the height of the predominant peak ^39^.

### Classification of recurrences and comparison of parasite genotype

Recrudescence was considered when parasites were detected by TBS or qPCR between days 4 and 28 after initiating antimalarial treatment. Relapse/reinfection was considered when parasitemia (TBS or qPCR) was detected at any time between days 29 and 120 after initiating treatment. 

For the comparison of parasite genotypes between isolates from the day of recurrence (detected between day 4 and 120 after initiating treatment) and those from the primary episode (detected on the day of admission or day 1), recurrences were considered genetically homologous when exactly the same alleles were detected in all microsatellites at both time points (primary episode and recurrence), and when allelic variants were detected in at least one microsatellite, they were classified as genetically heterologous recurrences ^21^^,^^40^.

### Relative quantification of immune mediator expression

First, total RNA extraction was performed from the leukocyte pellet homogenized in TRIzol (Invitrogen), maintained at refrigeration temperature and following the manufacturer’s instructions. Then, relative quantification of the expression of IL-13, IL-10, TNF-α, IFN-γ, IL- 8, TGF-β, Fox-P3, PD-L1 was performed with the commercial kit Express OneStep Superscript® qRT-PCR kit (Invitrogen), on the StepOnePlus real‐time PCR system (Applied Biosystems), with the protocol previously reported ^41^.

### Statistical analysis

Microsoft Excel v.10 and GraphPad Prism v.8 were used for analysis. No quantitative variable had parametric distribution, nor homogeneity of variances, according to Shapiro-Wilk and Levene, respectively; therefore, differences between unpaired groups (pregnant vs. non-pregnant and with recurrences vs. without recurrences) were evaluated with Mann-Whitney U and between paired groups (initial episode vs. recurrence) with Wilcoxon. Qualitative variables were compared with Chi-square. To analyze genetic diversity, the number of alleles per locus, the predominant allele, the percentage of polyclonal samples, and the expected heterozygosity (He) were calculated (He = [n/(n - 1) ] [1 - Σpi2]; n, number of alleles per locus and pi, frequency of allele i in the population). Statistical significance was always considered with p less than 0.05.

### Ethical considerations

This study was considered minimum risk (Resolution 8430 of 1993) and was approved by the Bioethics Committee of the Medical Research Institute of the Faculty of Medicine of the Universidad de Antioquia (act 012 of August 23, 2018).

## RESULTS

A total of 41 pregnant women (23 from Tierralta and 18 from Puerto Libertador) and 46 non-pregnant men and women (31 from Tierralta and 15 from Puerto Libertador) with uncomplicated vivax malaria, diagnosed by TBS and confirmed by qPCR, participated in this study. Pregnant women had an average age of 21 years (range: 14-42 years) and 22% (9/41) were in the first trimester, 54% (22/41) in the second trimester, and 24% (10/41) in the third trimester. In the non-pregnant group, 63% (29/46) were men and 37% (17/46) women, with an average age of 28 years (range: 8-84 years). Age and parasitemia both at the time of admission and recurrence were similar between pregnant and non-pregnant individuals (Table 1).

**Table 1:** Age and parasitemia levels in the primary episode and recurrence for the cohort of pregnant and non-pregnant patients with uncomplicated *P. vivax* malaria.

Variables	Pregnant (n=41)				Non-pregnant (n=46)				p (M-W)
	Min	Max	Med	IQR	Min	Max	Med	IQR	
Age (years)	14	42	19	17-23	8	84	21	14-43	0.670
Parasitemia (p/µL) in initial episode	63	33400	4180	1600-8032	720	29500	2403	1738-4815	0.296
Parasitemia (p/µL) in recurrent episode	286	21782	1670	1110-4841	1150	20530	2130	1173-15348	0.753

Min: minimum, Max: maximum, Med: median, p/uL: parasites/microliter, IQR: interquartile range, p (M-W): p-value from Mann-Whitney U test.

Among the 41 pregnant women studied, 4.9% (n=2) presented recrudescences (parasitemia detected between day 4 and 28 after initiating antimalarial treatment), while the remaining 95.1% (n=39) were classified as therapeutic successes (no parasitemia, nor malaria symptoms between days 4 and 28 after initiating treatment). In the non-pregnant cohort, 4.3% (n=2) had recrudescence and 95.7% (n=44) were therapeutic successes.

The 120-day follow-up allowed the detection of 21 relapses/ reinfections in the total of participants with therapeutic success (n=83), that is, detection of parasitemia between days 29 and 120 after initiating treatment, for a prevalence of relapses/reinfections of 25.3% (21/83). However, in the pregnant cohort, the prevalence of relapses/ reinfections was 43.6% (17/39), while in the non-pregnant cohort it was 9.1% (4/44).

Table 2 shows *P. vivax* genetic diversity data, with each microsatellite analyzed and in each cohort studied, both in the primary episode and in the recurrence. The following variables showed statistically significant differences between pregnant and non-pregnant individuals, both in the primary and recurrent episode: amplification success percentage, number of different alleles detected, and percentage of polyclonal infections. Furthermore, in the pregnant cohort, the following variables showed differences between the primary episode and the recurrence: number of different alleles detected, predominant allele, and percentage of polyclonal infections. In the non-pregnant cohort, significant differences were found in the number of alleles detected, the percentage of polyclonal infections, and the He between the primary and recurrent episode.

**Table 2:** Parasite genetic diversity detected with the three microsatellites in each studied cohort (pregnant and non-pregnant) and in each evaluated episode (primary episode and recurrence).

	Data per microsatellite								
	1.501	3.502	3.27	1.501	3.502	3.27	Median per cohort		D
Primary episode (enrollment, day 1)	Pregnant n=41			Non-pregnant n=46			Gestante	No gestante	
% amplification success	95	90	93	63	80	91	93	80	Yes
# of detected alleles	19	9	34	9	6	26	19	9	Yes
Predominant allele (bp)	106	144	336	85	144	288	-	-	No
% polyclonal samples	17	17	49	24	7	54	17	24	Yes
Ecpected heterozygosity (He)	0.95	0.93	0.98	0.92	0.80	0.95	0.95	0.92	No
Recurrent episode	Pregnant n=19			Non-pregnant n=6			Pregnant	Non-pregnant	
amplification success	95	79	95	83	83	100	95	83	Yes
# of detected alleles	8	8	23	2	3	8	8	3	Yes
Predominant allele^a^ (bp)	120	168	288 368	85	128 152 168	260 288	-	-	No
% polyclonal samples	37	11	58	0	17	33	37	17	Yes
Expected heterozygosity (He)	0.98	0.95	0.98	0.96	1.00	0.98	0.98	0.98	No

D: statistically significant difference (p < 0.05) between pregnant and non-pregnant women evaluated with Mann-Whitney U or Chi-square tests, as appropriate for quantitative or qualitative variables.

a In the recurrence, there was more than one predominant allele in microsatellites Pv3.502 and Pv3.27.

Additionally, the number of alleles detected was significantly higher in pregnant women than in non-pregnant individuals, both in the primary episode and in the recurrence, and in pregnant women, furthermore, the predominant allele detected in the primary episode was totally different from the predominant allele detected in the recurrence; while in non-pregnant individuals, some predominant alleles were the same between the primary episode and recurrence. However, no allele was exclusively detected in any cohort; all alleles were shared.

The total of malaria recurrences was more than double in pregnant women (46%, 19/41) than in non-pregnant individuals (13%, 6/46), for a ratio of 3.5:1. In pregnant women, recurrences were of the relapse/reinfection type in 89% of cases (17/19) and of the recrudescence type in 11% of cases (2/19), for a ratio of 8.1:1. While in non-pregnant individuals, recurrences were relapses/reinfections in 67% of cases (4/6) and recrudescences in 33% of cases (2/6), for a ratio of 2:1. Additionally, recrudescences had a ratio of 1:3 between pregnant and non-pregnant individuals (11%:33%) and relapses/reinfections had a ratio of 1.3:1 between pregnant and non-pregnant individuals (89%:67%) (Table 3).

**Table 3:** Frequency and type of malarial recurrences in the cohort of pregnant and non-pregnant women.

Type of recurrence	Pregnant	Non-pregnant	Total	p (χ²)
Recrudescence (between days 4 and 28)	2/19 (11%)	2/6 (33%)	4 (16%)	0.184
Relapse/reinfection (between days 29 and 120)	17/19 (89%)	4/6 (67%)	21 (84%)	0.004
Total recurrences	19/41 (46%)	6/46 (13%)	25 (29%)	<0.001

p (χ^2^): p-value of the Chi-square test for the comparison between pregnant and non-pregnant women

**Figure 1:**
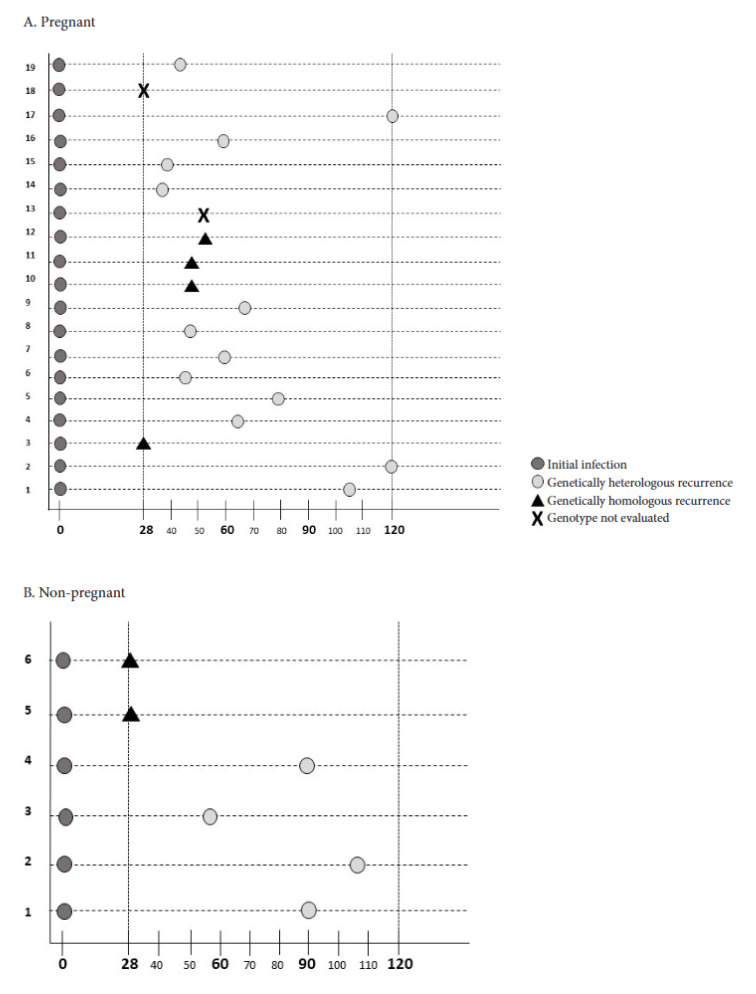
Timing of recurrence detection and comparison of the parasite genotype between admission and recurrence in each study cohort. A. Pregnant cohort: primary episode, not evaluated (2/19), heterologous recurrence (13/17), homologous recurrence (4/17). B. Non-pregnant cohort: primary episode, heterologous recurrence (4/6), homologous recurrence (2/6).

It was not possible to compare the parasite genotype between the primary and recurrent episode in two of the 19 pregnant women with malaria recurrence (11%), because adequate amplification of microsatellites was not obtained in one of the two episodes. In the remaining 17 pregnant women with recurrence, it was found that 23% had recurrences homologous to the initial episode and 77% had heterologous recurrences. For their part, in the six recurrent episodes detected in the non-pregnant cohort, it was found that 33% corresponded to homologous recurrences and 67% to heterologous recurrences. In summary, in both cohorts, heterologous recurrences are widely predominant, but the predominance is greater in pregnant women (3.3:1) than in non-pregnant individuals (2:1) (Figure 1).

Y-axis: represents the number of each volunteer with recurrence. X-axis: follow-up day

It was not possible to determine the expression of immune mediators in all samples, because in some cases it was not possible to collect the sample in TRIzol or adequate amplification could not be achieved. Table 4 shows the comparison of immune mediator expression between the initial episode and the recurrence of both cohorts, in the samples that were successfully analyzed. The only immune mediator that showed significant differences in expression between the primary episode and the recurrence was Fox-P3, and only in the pregnant cohort, who showed significantly higher levels of Fox-P3 in the initial episode, compared to the recurrence (Table 4).

**Table 4:** Comparison of the relative expression of immune mediators in maternal peripheral blood between enrollment and recurrence in each studied cohort.

Pregnant (n=34)						
Mediator (RU)	Enrollment (n=34)		Recurrence (n=13)		Enrollment / recurrence	p (M-W)
	Median	IQR	Median	IQR		
TNF-α	3.16	0.24-11	0.45	0.27-3.74	7.0	0.316
IFN-γ	8.27	1.82-16	6.67	4.79-7.76	1.2	0.249
IL-8	1.33	0.60-2.70	2.28	0.10-28	0.6	0.764
IL-13	19.81	5.80-32	13.62	2.55-42	1.5	0.650
TGF-β	6.51	0.52-21	1.98	1.01-29	3.3	0.986
IL-10	0.17	0.07-0.70	0.1	0.03-0.52	1.7	0.199
PD-L1	0.5	0.06-4.60	1.52	0.44-18	0.3	0.090
Fox-P3	0.7	0.29-2.08	0.26	0.09-0.80	2.7	0.020
Non-pregnant (n=30)						
Mediator (RU)	Enrollment (n=30)		Recurrence (n=2)		Enrollment / recurrence	p (M-W)
	Median	IQR	Median	IQR		
TNF-α	0.03	0.1-0.18	0.14	0.01-0.26	0.2	0.891
IFN-γ	3.21	0.80-7.01	2.77	1.03-4.51	1.2	0.896
IL-8	2.67	1.28-5.84	3.59	0.56-6.62	0.7	0.901
IL-13	1.88	0.64-3.20	1.24	0.76-1.72	1.5	0.541
TGF-β	0.02	0.01-0.17	0.23	0.01-0.44	0.1	0.904
IL-10	-	-	-	-		
PD-L1	80.32	38-190	76.06	17.52-135	1.1	0.640
Fox-P3	0.79	0.30-1.24	2.46	0.89-4.02	0.3	0.225

IQR: interquartile range, RU: relative units of expression. Enrollment/recurrence: ratio between the median at enrollment and recurrence, p (M-W): p-value from Mann-Whitney U test.

The comparison of immune mediator expression between pregnant and non-pregnant individuals both at admission (primary episode) and at recurrence is presented in Table 5. In summary, at admission, pregnant women showed significantly higher levels in the expression of TNF-α, IFN-ɣ, IL-8, IL-13, and TGF-β than non-pregnant individuals, while PD-L1 had higher expression in non-pregnant individuals than in pregnant women. In the recurrent episode, TGF-β expression was significantly higher in pregnant women than in non-pregnant individuals, and the other immune mediators had similar expression in both cohorts (Table 5).

**Table 5:** Comparison of the relative expression of immune mediators in peripheral blood between the cohort of pregnant and non-pregnant women at the time of enrollment and malarial recurrence.

	Enrollment (n=64)					
Mediator (RU)	Pregnant (n=34)		Non-pregnant (n=30)		Pregnant/non-pregnant	p (M-W)
	Median	IQR	Median	IQR		
TNF-α	0.87	0.18-3.92	0.03	0.01-0.26	29	<0.001
IFN-γ	8.25	6.50-9.97	3.37	0.79-7.11	2.4	<0.001
IL-8	5.20	1.20-9.16	1.61	0.72-2.35	3.2	0.006
IL-13	17.31	4.91-29	1.88	0.76-3.22	9.2	<0.001
TGF-β	4.22	0.27-16	0.02	0.01-0.17	210	<0.001
PD-L1	0.34	0.06-4.61	81.24	46-202	0.004	<0.000
Fox-P3	0.82	0.31-2.14	0.75	0.29-1.17	1.1	0.312
	Recurrence (n=15)					
Mediator (RU)	Pregnant (n=13)		Non-pregnant (n=2)		Pregnant/non-pregnant	p (M-W)
	Median	IQR	Median	IQR		
TNF-α	0.45	0.22-3.74	0.14	0.01-0.27	3.2	0.153
IFN-γ	6.16	5.02-7.52	2.77	1.03-4.52	2.2	0.121
IL-8	0.97	0.30-4.01	3.59	0.56-6.62	0.2	0.923
IL-13	14.69	3.51-46	1.24	0.76-1.72	11.8	0.153
TGF-β	2.48	0.97-35	0.23	0.01-0.45	10.8	0.044
PD-L1	2.80	0.41-19	76.06	18-135	0.03	0.181
Fox-P3	0.25	0.08-0.89	2.46	0.89-4.02	0.1	0.102

IQR: interquartile range, RU: relative units of expression, Pregnant/non-pregnant: ratio between the median of pregnant and non-pregnant women, p (M-W): p-value from Mann-Whitney U test.

To explore the association between the profile of immune mediators with the parasite genotype of the primary episode and the recurrence, data from participants who had complete paired information of parasite genotyping (the three microsatellites) and immune mediators at admission and recurrence were compared, which corresponded to only 9 of the 19 pregnant women with recurrence and 2 of the 6 non-pregnant individuals with recurrence. With this comparison, it was not possible to evidence any statistically significant difference in the expression of immune mediators between those who had homologous and heterologous recurrences (data not shown).

## DISCUSSION

This study compared *P. vivax* genotypes between the primary episode and the recurrence in the pregnant and non-pregnant population, and also explored the association between those parasite genotypes with the expression of proinflammatory and anti-inflammatory immune mediators. The findings showed that vivax malaria recurrences are considerably more frequent in pregnant women than in non-pregnant individuals, 46% versus 13%, respectively. In pregnant women, there was greater parasite genetic diversity, both in the primary episode and in the recurrent one, as well as a predominance of recurrences heterologous to the primary episode. Likewise, pregnant women showed higher levels of proinflammatory and anti-inflammatory immune mediators in the primary episode, and a significant decrease of Fox-P3 in the recurrence; while in non-pregnant individuals, immune mediators remained relatively stable between the initial episode and the recurrence.

These findings confirm the high genetic diversity of *P. vivax* in Colombia, both in the pregnant and non-pregnant population ^39^^,^^42^^-^^46^. Although the predominant alleles in each microsatellite studied were not exclusive to pregnant women, predominant alleles exclusive to the primary episode or the recurrence were found in pregnant women, which is consistent with previous Colombian studies in non-pregnant individuals, which show parasite haplotypes exclusive to recurrences ^29^.

In both cohorts, polyclonal infections were prevalent in primary episodes and recurrences, which coincides with previous Colombian studies in pregnant women ^44^^,^^46^ and non-pregnant individuals ^28^^,^^44^^,^^45^, and with international studies that highlight that *P. vivax* infections have high genetic complexity [25]. Pregnant women showed a higher frequency of polyclonal infections in the recurrence than in the initial episode, while non-pregnant individuals showed greater polyclonality in the primary episode than in the recurrence. This could be explained because pregnant women have a higher probability of presenting multiclonal recurrences due to not receiving primaquine, which acts on hypnozoites.

The frequency of relapses/reinfections detected in pregnant women was very high (41%, 17/41), even higher than reported in other Colombian studies: 29% with 120-day follow-up ^33^ and 18% to 24% with 180-day follow-up ^28^^,^^47^. The use of qPCR in addition to microscopy for infection detection in this study may explain that higher frequency of recurrences.

Heterologous recurrences were much more frequent than homologous ones, both in pregnant women and in non-pregnant individuals, which is consistent with the high number of polyclonal infections detected in both cohorts and coincides with other studies reporting that heterologous recurrences are generally polyclonal and occur in most endemic areas ^40^^,^^48^. These findings also agree with another Colombian study that included *P. vivax* patients resident in Turbo (endemic municipality) and Medellín (non-endemic municipality) and reported that isolates from recurrent infections were genetically different from isolates of the initial infection ^29^. But they contrast with another work carried out in Turbo that reported that 93% of recurrent infections by *P. vivax* were homologous ^28^.

To our knowledge, this is the first work exploring the association between the host immune profile with the parasite genotype in primary and recurrent episodes, both in pregnant and non-pregnant individuals in Colombia. No statistically significant differences were found in the level of expression of immune mediators between initial and recurrent infection, nor between homologous and heterologous recurrences. Therefore, there was no association of the host immune profile with plasmodial genotypes in initial and recurrent infection; but, perhaps, this is due to the small sample size available for analysis, as there are studies reporting the existence of differences between levels of immune mediators in recurrence, depending on whether it is genetically homologous or heterologous to the initial episode ^21^^,^^22^.

As a strength, the multifaceted approach to the problem in pregnant women is highlighted, having a cohort of non-pregnant individuals as a comparison group. Similarly, limitations such as the relatively small sample size, the low quantity of molecular markers used, and the lack of knowledge of the nutritional status of participants and their infection status with other parasites such as intestinal ones, which are conditions that can modulate the immune response, are accepted. However, these limitations do not invalidate the findings of the study, since comparisons are made between patients from the same region, who are exposed to similar socioeconomic conditions, generally precarious as in almost all malaria-endemic areas of the world, so it is expected that their nutritional status and infection with intestinal parasites would also be similar.

In conclusion, pregnant women are exposed to a high frequency of recurrences with high genetic variation, which may affect the development of effective protective immunity and generate adverse maternal-neonatal outcomes. This raises the need not only to strengthen actions for prevention of *P. vivax* malaria in pregnant women, but also to optimize antimalarial treatment and find alternatives to the hypnozoiticidal regimen currently available in almost all malaria-endemic areas.
