# Supplementary material for: Parasite genotypes and host immune mediators in primary and recurrent episodes of vivax Malaria in Colombia
Source: Rev Peru Med Exp Salud Publica. 2025 Dec 13;42(4):389–99. doi: 10.17843/rpmesp.2025.424.14865 (PMC12879981; doi:10.17843/rpmesp.2025.424.14865)
Supplement: Translation

# Translation: Genotipos parasitarios y mediadores inmunes del hospedero en episodios primarios y recurrentes de la malaria vivax en Colombia

**Authors:** Yesica Yamile Duque-Isaza, Jaime Carmona-Fonseca, Eliana María Arango-Flórez

**Affiliations:** 1 Grupo Salud y Comunidad-César Uribe Piedrahíta, Facultad de Medicina, Universidad de Antioquia, Medellín, Colombia.; 2 GEINCRO, Facultad de Ciencias de la Salud, Fundación Universitaria San Martín, Sabaneta, Colombia.

**Keywords:** Malaria, Plasmodium vivax, Recurrencia, Embarazo, Genotipos, Citoquinas

## Abstract

**Objetivos.:**

Identificar los genotipos de *Plasmodium vivax* en población gestante, y no gestante y explorar su asociación con mediadores inmunes.

**Materiales y métodos.:**

Se siguieron durante 120 días dos cohortes de pacientes con malaria vivax no complicada en Puerto Libertador y Tierralta, Córdoba, Colombia: 41 gestantes y 46 no gestantes, todas tratadas con el tratamiento estándar. Se compararon los genotipos parasitarios (microsatélites Pv3.27, Pv3.502 y Pv1.501) y la expresión de mediadores inmunes del hospedero (IL-13, IL-10, TNF-alfa, IFN-gamma, IL-8, TGF-beta, Fox-P3, PD-L1) entre infecciones primarias y recurrentes.

**Resultados.:**

La frecuencia de recurrencias fue mayor en gestantes (41,6%), que en no gestantes (8,7%). La diversidad genética parasitaria fue mayor en gestantes, aunque sin alelos exclusivos. Las recurrencias fueron genéticamente homólogas (mismos alelos) solo en 23% y 33% de los casos en gestante y no gestantes, respectivamente. En cuanto a los mediadores inmunes, solo la expresión de Fox-P3 varió significativamente en gestantes, con mayor expresión en el episodio primario que en el recurrente.

**Conclusiones.:**

Las mujeres embarazadas están expuestas a una alta frecuencia de recurrencias de malaria vivax, con alta variación genética del parásito, lo que puede afectar el desarrollo de inmunidad protectora efectiva y favorecer desenlaces obstétricos adversos, tanto maternos como neonatales. Estos hallazgos plantean la necesidad de fortalecer y optimizar las acciones de prevención y tratamiento de la malaria *vivax* durante la gestación.

## INTRODUCCIÓN

La infección por *Plasmodium vivax* durante el embarazo se asocia con enfermedad en la mujer (malaria gestacional), daño placentario (malaria placentaria) ^1^^)^ y desenlaces maternos y perinatales adversos ^2^^,^^3^. Se estima que, cada año, más de 90 millones de gestaciones en el mundo están en riesgo de infección por *P. vivax*^4^. En particular, en el continente americano, esta especie es responsable de más del 80% de los casos de malaria ^5^, con reportes del noroccidente de Colombia que documentan una frecuencia de malaria gestacional del 35,8% y malaria placentaria entre 20,9% y 27,7% ^6^^,^^7^.

Los estudios que aporten en el conocimiento de la genética y la patogenicidad del parasito, así como en la respuesta inmune del hospedero, son indispensables para comprender los procesos fisiopatológicos de esta especie. En general el conocimiento sobre *P. vivax* es mucho menos avanzado que el de *P. falciparum*. No obstante, los reportes disponibles no dejan dudas sobre el papel patogénico de *P. vivax* durante la gestación ni sobre los efectos mórbidos que produce en las gestantes y sus hijos ^1^^-^^3^^,^^8^^,^^9^.

Todas las especies de *Plasmodium* pueden causar recurrencias maláricas, entendidas como la reaparición de la parasitemia, con o sin síntomas asociados, luego de haber recibido tratamiento antimalárico específico ^10^^,^^11^. Esas recurrencias pueden ser de tres tipos: A) recrudescencia, causada por la eliminación incompleta de los estadios eritrocíticos (falla terapéutica). Suele aparecer dentro de los 28 días de iniciado el tratamiento y puede ser causada por cualquier especie de *Plasmodium*^10^^,^^12^. B) Reinfección, generada por la inoculación de nuevos esporozoítos por el vector. Ocurre después de los 28 días de iniciado el tratamiento en pacientes con curación completa del episodio inicial, y puede ser causada por cualquier especie ^10^^,^^12^^,^^13^. C) Recaída, causada por la activación de los hipnozoítos (estadios hepáticos latentes) y aparece después de los 28 días de iniciado el tratamiento para la malaria por *P. vivax* o *P. ovale,* en paciente que tuvieron una resolución completa del episodio primario ^10^^,^^12^^-^^14^.

Otras fuentes de recaídas sugeridas son estadios eritrocíticos secuestrados en bazo y médula ósea ^15^, esporozoítos que sobreviven en piel y merozoítos presentes en el sistema linfático ^16^. En pacientes provenientes de zonas no endémicas, la diferenciación entre recrudescencia, reinfección y recaída puede realizarse mediante seguimiento clínico y parasitológico. No obstante, en residentes de zonas endémicas, esta diferenciación es limitada, ya que actualmente no se dispone de herramientas accesibles que permitan distinguir de manera confiable entre recaídas y reinfecciones ^17^.

La primaquina (8-aminoquinolina) como hipnozoítocida, en combinación con la cloroquina (4-aminoquinolina), que actúa como esquizonticida sanguíneo, constituye al tratamiento indicado para curar la malaria *vivax* y prevenir las recaídas en Colombia y en la mayoría de las zonas endémicas. Sin embargo, la primaquina está contraindicada durante el embarazo y la lactancia debido al riesgo de hemólisis dosis-dependiente en fetos y recién nacidos con deficiencia no diagnosticada de glucosa-6-fosfato deshidrogenasa ^(^^18^^,^^19^.

La cantidad de recurrencias por *P. vivax* podria estar influenciada por la inmunidad adquirida durante el episodio primario y por la historia inmunológica de episodios anteriores ^20^. Los pacientes adquieren inmunidad natural después de la infección primaria, y estudios experimentales han demostrado que la protección inmune es específica de clon ^21^. Sin embargo, la información disponible sobre la respuesta inmune en las recurrencias maláricas es escasa ^22^^,^^23^. En Tailandia se observó que tanto las citoquinas proinflamatorias como las antiinflamatorias presentaban niveles más elevados en pacientes con *P. vivax* que en individuos sanos. Además, en pacientes con recaídas homólogas o heterólogas, los niveles de IL-1β fueron más altos durante la infección primaria, mientras que los niveles de IL-6 e IL-10 fueron mayores en las recaídas heterólogas que en las homólogas ^21^.

La diversidad genética de *P. vivax* es mucho mayor que la de *P. falciparum*^24^^,^^25^. Genotípicamente, las recurrencias maláricas pueden ser homólogas o heterólogas: en las homólogas, los genotipos parasitarios del episodio primario y la recurrencia son exactamente iguales ^26^^,^^27^, mientras que en las heterólogas se encuentran genotipos parasitarios diferentes en el episodio primario y en la recurrencia ^27^. En Colombia pocos estudios han comparado los genotipos de *P. vivax* entre la infección primaria y la recurrencia ^28^^,^^29^, y ninguno ha sido realizado en población gestante. La respuesta inmune del hospedero humano a la infección plasmodial es muy compleja, y cuando ocurre durante la gestación, esta complejidad es aún mayor debido a los cambios hormonales e inmunológicos que ocurren durante el embarazo, donde es necesario mantener un balance entre la respuesta inmune proinflamatoria y antiinflamatoria ^30^.

Este estudio tuvo como objetivo comparar los genotipos de *P. vivax* entre el episodio primario y la recurrencia en población gestante y no gestante, así como explorar la asociación entre los genotipos parasitarios presentes en esos episodios maláricos con la expresión de mediadores inmunes proinflamatorios y antiinflamatorios en ambos grupos.

**MENSAJE CLAVE**

Motivación del estudio. La malaria vivax constituye un reto relevante en salud pública en Colombia, especialmente en mujeres gestantes, más propensas a recurrencias y complicaciones. Sin embargo, la diversidad genética del parásito en infecciones primarias y recurrentes, así como su relación con la respuesta inmunitaria del hospedero, ha sido escasamente estudiada.

Principales hallazgos. Las gestantes presentaron mayor frecuencia de recurrencias (41%) y una mayor diversidad genética del parasito en comparación con las no gestantes. Asimismo, se observó una mayor expresión de Fox-P3 durante la infección primaria de gestantes, lo que puede modular la inmunidad protectora.

Implicaciones en salud pública. Fortalecer la prevención y control de la malaria por *P. vivax* en el embarazo es crucial para disminuir recurrencias y mejorar los desenlaces maternos y neonatales.

## MATERIALES Y MÉTODOS

### Lugar y población de estudio

Esta investigación se ejecutó en Puerto Libertador (7°53′17″N 75°40′25″W) y Tierralta (8°10′22″N 76°03′34″W), en el departamento de Córdoba, Colombia. Ambos pertenecen al segundo foco de transmisión de malaria en Colombia (Urabá-Bajo Cauca-Sinú-San Jorge), donde *P. falciparum* y *P. vivax* cocirculan, aunque *P. vivax* predomina, con un 65% de los casos ^31^^,^^32^. Aproximadamente el 51% de los casos de malaria vivax reportados en Colombia provienen de esta región. Entre los años 2000 y 2023, se registraron 674 294 casos acumulados, con un índice anual de *P. vivax* (número de casos por cada 1000 habitantes) de 13,9 ^32^.

### Diseño del estudio

Se llevó a cabo un estudio descriptivo, prospectivo y longitudinal, con dos cohortes: 41 mujeres gestantes y 46 hombres y mujeres no gestantes, todos voluntarios con diagnóstico de malaria no complicada causada exclusivamente por *P. vivax.* La infección fue detectada mediante gota gruesa (GG) y confirmada por PCR cuantitativa en tiempo real (qPCR). La inclusión de participantes fue progresiva entre 2016 y 2019, tanto en el puesto de diagnóstico de malaria del hospital local, como en la consulta prenatal.

### Criterios de selección

*Criterios de inclusión*

Se invitaron a participar mujeres embarazadas de cualquier edad gestacional, así como hombres y mujeres no gestantes, que presentaron infección única por *P. vivax* confirmada por GG y qPCR, que fueron clasificados como casos de malaria no complicada y que consultaron voluntariamente en el hospital local entre 2016-2019. Todos los participantes consintieron su participación voluntaria mediante la firma del consentimiento informado y en casos de menores de edad, también se firmó el asentimiento informado.

*Criterios de exclusión*

Se excluyeron aquellas gestantes con antecedentes de comorbilidades compatibles con el síndrome TORCHS, según registro en la historia clínica, así como los pacientes (gestantes o no gestantes) que hubieran recibido algún antimalárico distinto del tratamiento estándar prescrito en el hospital local. También se excluyeron aquellos que retiraron el consentimiento, desarrollaron alguna complicación malárica durante el seguimiento o reportaron embarazo en el transcurso del estudio dentro de la cohorte de no gestantes.

### Tratamiento antimalárico

El tratamiento suministrado a los voluntarios de este estudio fue el recomendado por la Organización Mundial de la Salud (OMS) y las autoridades sanitarias de Colombia para la malaria no complicada por *P. vivax*. En pacientes no gestantes, se administró la combinación de cloroquina durante tres días (dosis total de 25 mg/kg) más primaquina durante 14 días (dosis diaria de 0,25 mg/kg). En gestantes, se prescribió únicamente cloroquina (dosis total de 25 mg/kg dividida en tres días), debido a la contraindicación de la primaquina durante el embarazo ^(^^18^^,^^33^. El tratamiento fue entregado por el personal del puesto de diagnóstico de malaria del hospital local, y su administración fue no supervisada.

### Recolección de muestras y seguimiento

El día de ingreso al estudio (día 1), antes de la administración de la primera dosis del tratamiento antimalárico, se realizó venopunción a cada voluntario para obtener una muestra de sangre periférica, para preparar: a) dos láminas de GG para el diagnóstico microscópico de la malaria; b) tres círculos de papel de filtro Whatman® No. 3 para la extracción de ADN para la confirmación de la infección única por *P. vivax* mediante qPCR y para la genotipificación de los parásitos con los microsatélites; c) dos viales, cada uno con 100 µL del paquete leucocitario mezclado con 900 µL de TRIzol (Invitrogen), conservados en nitrógeno líquido hasta su uso en la extracción del ARN para la cuantificación de la expresión de los mediadores inmunes.

El seguimiento para la detección de las recurrencias se realizó en los días 28, 60, 90 y 120 después del inicio del tratamiento antimalárico. En cada seguimiento se evaluó la sintomatología malárica y se efectuó venopunción para colectar las mismas muestras obtenidas el día de ingreso. Las visitas no programadas de los voluntarios entre los días 2 y 120, también fueron consideradas para la detección de las recurrencias; es decir, si algún voluntario presentó GG positiva o sintomatología malárica en días diferentes al 28, 60, 90 o 120, también se consideró como recurrencia malárica y, ese día, se colectaron las mismas muestras de sangre del día de ingreso.

### Diagnóstico de la infección plasmodial

Las láminas de GG fueron teñidas con colorante Field y leídas por un técnico microscopista experimentado del hospital local, siguiendo las recomendaciones de la OMS ^34^. Para el cálculo de la parasitemia se realizó un recuento de parásitos en un total de 200 leucocitos y se usó la constante de 8000 leucocitos/µL.

La confirmación de la infección única por *P. vivax* se hizo mediante qPCR, con el uso del protocolo y los cebadores y sondas para el gen 18S, que fueron diseñados por investigadores de Edmonton, Canadá ^35^. La extracción del ADN a partir del papel de filtro se hizo mediante el método de Saponina-Chelex previamente descrito por otros investigadores ^(^^36^.

### Genotipificación de parásitos

La amplificación de los microsatélites Pv3,27, Pv3,502 y Pv1,501 se hizo por PCR semianidada según Koepfli y colaboradores ^(^^37^, con algunas modificaciones: 1X PCR buffer (Qiagen), 3 mM (Pv3,27) o 3,5 mM (Pv3,502, Pv1,501) de MgCl_2_ (Qiagen), 200 μM de cada desoxinucleótido trifosfato (dNTP) (Takara Bio), 0,25 μM de cada cebador y 0,067 IU/µL de Taq DNA polimerasa recombinante (Fermentas). Para confirmar la adecuada amplificación, se corrieron 2 µL de cada producto en electroforesis en gel de agarosa al 2%, teñido con GelRed™ (Biotium) y se visualizaron las bandas en transiluminador UV.

El análisis del tamaño de los productos amplificados se realizó por electroforesis capilar en un analizador genético 3500 (Hitachi Applied Biosystems), con el estándar interno GeneScan 600 LIZ (Applied Biosystems) y las condiciones de fábrica para microsatélites. Los productos de PCR se diluyeron en agua Milli-Q, 1:10 (Pv3,502) o 1:20 (Pv1,501 y Pv3,27). Los datos de la electroforesis se analizaron con GeneMapper ID-X Software v1.5 (Applied Biosystems) y los electroferogramas se examinaron visualmente. Los picos por encima de 300 unidades de fluorescencia relativas (UFR), se consideraron verdaderas amplificaciones. Los alelos se agruparon manualmente según su tamaño, así: grupos de 4 pb para Pv3,27; 7 pb para Pv1,501 y 8 pb para Pv3,502 ^(^^38^. Se estableció infección policlonal cuando en un locus se observó más de un pico y la altura del pico menor era superior a 33% de la altura del pico predominante ^(^^39^.

### Clasificación de recurrencias y comparación del genotipo parasitario

Se consideró recrudescencia cuando se detectaron parásitos por GG o qPCR entre los días 4 y 28 después de iniciado el tratamiento antimalárico. Se consideró recaída/reinfección cuando se detectó parasitemia (GG o qPCR) en algún momento entre los días 29 y 120 después de iniciado el tratamiento.

Para la comparación de los genotipos parasitarios entre los aislamientos del día de la recurrencia (detectados entre el día 4 y 120 después de iniciado el tratamiento) y los del episodio primario (detectados el día de ingreso o día 1), se consideraron recurrencias genéticamente homólogas, cuando se detectaron exactamente los mismos alelos en todos los microsatélites en ambos momentos (episodio primario y recurrencia), y cuando se detectaron variantes alélicas en al menos un microsatélite, se clasificaron como recurrencias genéticamente heterólogas ^21^^,^^40^.

### Cuantificación relativa de la expresión de mediadores inmunes

Primero, se hizo la extracción del ARN total a partir del paquete leucocitario homogenizado en TRIzol (Invitrogen), mantenido a temperatura de refrigeración y siguiendo las instrucciones del fabricante. Luego, se hizo la cuantificación relativa de la expresión de IL-13, IL-10, TNF-α, IFN-γ, IL-8, TGF-β, Fox-P3, PD- L1 con el estuche comercial Express OneStep Superscript® qRT-PCR kit (Invitrogen), en el sistema StepOnePlus real‐time PCR (Applied Biosystems), con el protocolo previamente reportado ^41^.

### Análisis estadístico

Se utilizó Microsoft Excel v.10 y GraphPad Prism v.8 para el análisis. Ninguna variable cuantitativa tuvo distribución paramétrica, ni homogeneidad de varianzas, según Shapiro-Wilk y Levene, respectivamente, por lo tanto, las diferencias entre los grupos no pareados (gestantes *vs.* no gestantes y con recurrencias *vs.* sin recurrencias) se evaluaron con U Mann-Whitney y entre los grupos pareados (episodio inicial *vs.* recurrencia) con Wilcoxon. Las variables cualitativas se compararon con Chi-cuadrado. Para analizar la diversidad genética, se calculó el número de alelos por locus, el alelo predominante, el porcentaje de muestras policlonales y la heterocigosidad esperada (He) (He = [n/(n - 1)] [1 - Σpi2]; n, número de alelos por locus y pi, frecuencia del alelo i en la población). La significancia estadística siempre se consideró con p menor de 0,05.

### Consideraciones éticas

Este estudio se consideró de riesgo mínimo (Resolución 8430 de 1993) y fue aprobado por el Comité de Bioética del Instituto de Investigaciones Médicas de la Facultad de Medicina de la Universidad de Antioquia (acta 012 del 23 de agosto de 2018).

## RESULTADOS

Un total de 41 mujeres gestantes (23 de Tierralta y 18 de Puerto Libertador) y 46 hombres y mujeres no gestantes (31 de Tierralta y 15 de Puerto Libertador) con malaria vivax no complicada, diagnosticada por GG y confirmada por qPCR, participaron en este estudio. Las gestantes tuvieron una edad promedio de 21 años (rango: 14-42 años) y 22% (9/41) se encontraban en el primer trimestre, 54% (22/41) en segundo trimestre y 24% (10/41) en tercer trimestre. En el grupo de no gestantes, 63% (29/46) eran hombres y 37% (17/46) mujeres, con edad promedio de 28 años (rango: 8-84 años). La edad y la parasitemia tanto en el momento del ingreso como de la recurrencia fue similar entre gestantes y no gestantes (tabla 1).

**Tabla 1:** Edad y nivel de parasitemia en el episodio primario y la recurrencia de la cohorte de gestantes y no gestantes con malaria no complicada por *P. vivax.*

Variables	Gestantes (n=41)				No gestantes (n=46)				p (M-W)
	Min	Max	Med	RIC	Min	Max	Med	RIC	
Edad (años)	14	42	19	17-23	8	84	21	14-43	0,670
Parasitemia (p/µL) en episodio inicial	63	33400	4180	1600-8032	720	29500	2403	1738-4815	0,296
Parasitemia (p/µL) en episodio recurrente	286	21782	1670	1110-4841	1150	20530	2130	1173-15348	0,753

Min: mínimo, Max: máximo, Med: mediana, p/uL: parasitos/microlitro, RIC: rango intercuartilico, p (M-W): valor de p de prueba U de Mann-Whitney.

Entre las 41 gestantes estudiadas, 4,9% (n=2) presentaron recrudescencias (parasitemia detectada entre el día 4 y 28 después de iniciado el tratamiento antimalárico), mientras que el restante 95,1% (n=39) fueron clasificados como éxitos terapéuticos (sin parasitemia, ni síntomas maláricos entre los días 4 y 28 después de iniciado el tratamiento). En la cohorte de no gestantes, 4,3% (n=2) tuvieron recrudescencia y 95,7% (n=44) fueron éxitos terapéuticos.

El seguimiento de 120 días permitió detectar 21 recaídas/reinfecciones en el total de participantes con éxito terapéutico (n=83), es decir, detección de parasitemia entre los días 29 y 120 después de iniciado el tratamiento, para una prevalencia de recaídas/reinfecciones de 25,3% (21/83). Sin embargo, en la cohorte de gestantes la prevalencia de recaídas/reinfecciones fue de 43,6% (17/39), mientras que en la cohorte de no gestantes fue de 9,1% (4/44).

En la tabla 2 muestra los datos de diversidad genética de *P. vivax,* con cada microsatélite analizado y en cada cohorte estudiada, tanto en el episodio primario como en la recurrencia. Las siguientes variables mostraron diferencias estadísticamente significativas entre gestantes y no gestantes, tanto en el episodio primario como en el recurrente: porcentaje de éxito de amplificación, número de alelos diferentes detectados y porcentaje de infecciones policlonales. Además, en la cohorte de gestantes, las siguientes variables mostraron diferencias entre el episodio primario y la recurrencia: número de alelos diferentes detectados, alelo predominante y porcentaje de infecciones policlonales. En la cohorte de no gestantes se encontraron diferencias significativas en el número de alelos detectados, el porcentaje de infecciones policlonales y la He entre el episodio primario y el recurrente.

**Tabla 2:** Diversidad genética parasitaria detectada con los tres microsatélites en cada cohorte estudiada (gestantes y no gestantes) y en cada episodio evaluado (episodio primario y recurrencia).

	Datos por microsatélite								
	1,501	3,502	3,27	1,501	3,502	3,27	Mediana por cohorte		D
Episodio primario (ingreso, día 1)	Gestantes n=41			No gestantes n=46			Gestante	No gestante	
% de éxito de amplificación	95	90	93	63	80	91	93	80	Sí
# de alelos detectados	19	9	34	9	6	26	19	9	Sí
Alelo predominante (pb)	106	144	336	85	144	288	-	-	No
% de muestras policlonales	17	17	49	24	7	54	17	24	Sí
Heterocigosidad esperada (He)	0,95	0,93	0,98	0,92	0,80	0,95	0,95	0,92	No
Episodio recurrente	Gestantes n=19			No gestantes n=6			Gestante	No gestante	
% de éxito de amplificación	95	79	95	83	83	100	95	83	Sí
# de alelos detectados	8	8	23	2	3	8	8	3	Sí
Alelo predominante^a^ (pb)	120	168	288 368	85	128 152 168	260 288	-	-	No
% de muestras policlonales	37	11	58	0	17	33	37	17	Sí
Heterocigosidad esperada (He)	0,98	0,95	0,98	0,96	1,00	0,98	0,98	0,98	No

D: diferencia estadísticamente significativa (p<0,05) entre gestantes y no gestantes evaluada con las pruebas U de Mann-Whitney o Chi al cuadrado, según correspondiera a una variable cuantitativa o cualitativa.

a En la recurrencia hubo más de un alelo predominante en los microsatélites Pv3,502 y Pv3,27.

Adicionalmente, el número de alelos detectados fue significativamente mayor en las gestantes que en los no gestantes, tanto en el episodio primario como en la recurrencia, y en las gestantes, además, el alelo predominante detectado en el episodio primario fue totalmente diferente al alelo predominante detectado en la recurrencia; mientras que, en los no gestantes, algunos alelos predominantes fueron iguales entre episodio primario y recurrencia. Sin embargo, ningún alelo fue exclusivamente detectado en alguna cohorte, todos los alelos fueron compartidos.

El total de recurrencias maláricas fue más del doble en las gestantes (46%, 19/41) que en no gestantes (13%, 6/46), para una razón de 3,5:1. En las gestantes, las recurrencias fueron de tipo recaída/reinfección en 89% de los casos (17/19) y del tipo recrudescencia en 11% de los casos (2/19), para una razón de 8,1:1. Mientras que en no gestantes, las recurrencias fueron recaídas/reinfecciones en 67% de los casos (4/6) y recrudescencias en 33% de los casos (2/6), para una razón de 2:1. Adicionalmente, las recrudescencias tuvieron una razón de 1:3 entre gestantes y no gestantes (11%:33%) y las recaídas/reinfecciones tuvieron una razón de 1,3:1 entre gestantes y no gestantes (89%:67%) (tabla 3).

**Tabla 3:** Frecuencia y tipo de recurrencias maláricas en la cohorte de gestantes y no gestantes.

Tipo de recurrencia	Gestantes	No gestantes	Total	p (χ²)
Recrudescencia (entre días 4 y 28)	2/19 (11%)	2/6 (33%)	4 (16%)	0,184
Recaída/reinfección (entre días 29 al 120)	17/19 (89%)	4/6 (67%)	21 (84%)	0,004
Recurrencias totales	19/41 (46%)	6/46 (13%)	25 (29%)	<0,001

p (χ²): valor p de la prueba Chi al cuadrado para la comparación entre gestantes y no gestantes.

No fue posible comparar el genotipo parasitario entre el episodio primario y el recurrente en dos de las 19 gestantes con recurrencia malárica (11%), porque no se obtuvo una adecuada amplificación de los microsatélites en alguno de los dos episodios. En las restantes 17 gestantes con recurrencia se encontró que 23% tenían recurrencias homólogas al episodio inicial y 77% tuvieron recurrencias heterólogas. Por su parte, en los seis episodios recurrentes detectados en la cohorte de no gestantes, se encontró que 33% correspondían a recurrencias homólogas y 67% a recurrencias heterólogas. En resumen, en ambas cohortes las recurrencias heterólogas son ampliamente predominantes, pero el predominio es mayor en gestantes (3,3:1) que en no gestantes (2:1) (figura 1).

**Figura 1:** Momento de detección de la recurrencia y comparación del genotipo parasitario entre el ingreso y la recurrencia en cada cohorte estudiada A. Cohorte de gestantes: episodio primario, no evaluada (2/19), recurrencia heteróloga (13/17), recurrencia homóloga (4/17). B. cohorte de no gestan-tes: Episodio primario, recurrencia heteróloga (4/6), recurrencia homóloga (2/6)

Eje Y: representa el número de cada voluntario con recurrencia. Eje X: día de seguimiento

No se pudo determinar la expresión de los mediadores inmunes en la totalidad de las muestras, porque en algunos casos no se logró colectar la muestra en TRIzol o no se pudo lograr una amplificación adecuada. En la tabla 4 se muestran la comparación de la expresión de los mediadores inmunes entre el episodio inicial y la recurrencia de ambas cohortes, en las muestras que se lograron analizar. El único mediador inmune que mostró diferencias significativas en la expresión entre el episodio primario y la recurrencia fue Fox-P3 y solamente en la cohorte de gestantes, quienes mostraron niveles significativamente mayores de Fox-P3 en el episodio inicial, comparado con la recurrencia (tabla 4).

**Tabla 4:** Comparación de la expresión relativa de los mediadores inmunes en sangre periférica maternal entre el ingreso y la recurrencia en cada cohorte estudiada.

Gestantes (n=34)						
Mediador (UR)	Ingreso (n=34)		Recurrencia (n=13)		Ingreso / recurrencia	p (M-W)
	Mediana	RIC	Mediana	RIC		
TNF-α	3,16	0,24-11	0,45	0,27-3,74	7,0	0,316
IFN-γ	8,27	1,82-16	6,67	4,79-7,76	1,2	0,249
IL-8	1,33	0,60-2,70	2,28	0,10-28	0,6	0,764
IL-13	19,81	5,80-32	13,62	2,55-42	1,5	0,650
TGF-β	6,51	0,52-21	1,98	1,01-29	3,3	0,986
IL-10	0,17	0,07-0,70	0,1	0,03-0,52	1,7	0,199
PD-L1	0,5	0,06-4,60	1,52	0,44-18	0,3	0,090
Fox-P3	0,7	0,29-2,08	0,26	0,09-0,80	2,7	0,020
No gestantes (n=30)						
Mediador (UR)	Ingreso (n=30)		Recurrencia (n=2)		Ingreso / recurrencia	p (M-W)
	Mediana	RIC	Mediana	RIC		
TNF-α	0,03	0.,1-0,18	0,14	0,01-0,26	0,2	0,891
IFN-γ	3,21	0,80-7,01	2,77	1,03-4,51	1,2	0,896
IL-8	2,67	1,28-5,84	3,59	0,56-6,62	0,7	0,901
IL-13	1,88	0,64-3,20	1,24	0,76-1,72	1,5	0,541
TGF-β	0,02	0,01-0,17	0,23	0,01-0,44	0,1	0,904
IL-10	-	-	-	-		
PD-L1	80,32	38-190	76,06	17,52-135	1,1	0,640
Fox-P3	0,79	0,30-1,24	2,46	0,89-4,02	0,3	0,225

RIC: rango intercuartílico, UR: unidades relativas de expresión. Ingreso/recurrencia: razón entre la mediana del ingreso y recurrencia, p (M-W): valor p de la prueba U de Mann-Whitney.

La comparación de la expresión de los mediadores inmunes entre gestantes y no gestantes tanto en el ingreso (episodio primario) como en la recurrencia se presentan en la tabla 5. En resumen, en el ingreso las gestantes mostraron niveles significativamente mayores en la expresión de TNF-α, IFN-ɣ, IL-8, IL-13 y TGF-β que en no gestantes, mientras PD-L1 tuvo mayor expresión los no gestantes que en gestantes. En el episodio recurrente, la expresión de TGF-β fue significativamente mayor en las gestantes que en las no gestantes y los demás mediadores inmunes tuvieron una expresión similar en ambas cohortes (tabla 5).

**Tabla 5:** Comparación de la expresión relativa de los mediadores inmunes en sangre periférica entre la cohorte de gestantes y no gestantes, en el momento del ingreso y la recurrencia malárica.

	Ingreso (n=64)					
Mediador (UR)	Gestantes (n=34)		No gestantes (n=30)		Gestante/no gestante	p (M-W)
	Mediana	RIC	Mediana	RIC		
TNF-α	0,87	0,18-3,92	0,03	0,01-0,26	29	<0,001
IFN-γ	8,25	6,50-9,97	3,37	0,79-7,11	2,4	<0,001
IL-8	5,20	1,20-9,16	1,61	0,72-2,35	3,2	0,006
IL-13	17,31	4,91-29	1,88	0,76-3,22	9,2	<0,001
TGF-β	4,22	0,27-16	0,02	0,01-0,17	210	<0,001
PD-L1	0,34	0,06-4,61	81,24	46-202	0,004	<0,000
Fox-P3	0,82	0,31-2,14	0,75	0,29-1,17	1,1	0,312
	Recurrencia (n=15)					
Mediador (UR)	Gestantes (n=13)		No gestantes (n=2)		Gestante/no gestante	p (M-W)
	Mediana	RIC	Mediana	RIC		
TNF-α	0,45	0,22-3,74	0,14	0,01-0,27	3,2	0,153
IFN-γ	6,16	5,02-7,52	2,77	1,03-4,52	2,2	0,121
IL-8	0,97	0,30-4,01	3,59	0,56-6,62	0,2	0,923
IL-13	14,69	3,51-46	1,24	0,76-1,72	11,8	0,153
TGF-β	2,48	0,97-35	0,23	0,01-0,45	10,8	0,044
PD-L1	2,80	0,41-19	76,06	18-135	0,03	0,181
Fox-P3	0,25	0,08-0,89	2,46	0,89-4,02	0,1	0,102

RIC: rango intercuartílico, UR: unidades relativas de expresión, Gestante/no gestante: razón entre mediana de las gestantes y las no gestantes, p (M-W): valor p de la prueba U Mann-Whitney.

Para explorar la asociación entre el perfil de mediadores inmunes con el genotipo parasitario del episodio primario y la recurrencia, se compararon los datos de los participantes que tenían la información completa pareada de la genotipificación parasitaria (los tres microsatélites) y de los mediadores inmunes en el ingreso y la recurrencia, que correspondió solamente a 9 de las 19 gestantes con recurrencia y 2 de los 6 no gestantes con recurrencia. Con esta comparación no se pudo evidenciar ninguna diferencia estadísticamente significativa entre en la expresión de los mediadores inmunes entre aquellos que tenía recurrencias homólogas y heterólogas (datos no mostrados).

## DISCUSIÓN

Este estudio comparó los genotipos de *P. vivax* entre el episodio primario y la recurrencia en población gestante, y no gestante y; asimismo, exploró la asociación entre esos genotipos parasitarios con la expresión de mediadores inmunes proinflamatorios y antiinflamatorios. Los hallazgos mostraron que las recurrencias de la malaria vivax son considerablemente más frecuentes en gestantes que en no gestantes, 46% frente a 13%, respectivamente. En las gestantes hubo mayor diversidad genética parasitaria, tanto en el episodio primario como en el recurrente, así como un predominio de recurrencias heterólogas al episodio primario. Asimismo, las gestantes mostraron mayores niveles de mediadores inmunes proinflamatorios y antiinflamatorios en el episodio primario, y un descenso significativo de Fox-P3 en la recurrencia; mientras que en los no gestantes, los mediadores inmunes permanecieron relativamente estables entre el episodio inicial y la recurrencia.

Estos hallazgos confirman la alta diversidad genética de *P. vivax* en Colombia, tanto en población gestante como no gestante ^39^^,^^42^^-^^46^. A pesar de que los alelos predominantes en cada microsatélite estudiado no fueron exclusivos de las gestantes, si se encontró en las gestantes alelos predominantes exclusivos del episodio primario o de la recurrencia, lo cual es consistente con estudios colombianos previos en no gestantes, que muestran haplotipos parasitarios exclusivos de las recurrencias ^29^.

En ambas cohortes fueron prevalentes las infecciones policlonales en episodios primarios y recurrencias, lo que coincide con estudios colombianos previos en gestantes ^44^^,^^46^^)^ y no gestantes ^(^^28^^,^^44^^,^^45^, y con estudios internacionales que resaltan que las infecciones por *P. vivax* tiene alta complejidad genética [25]. Las gestantes mostraron mayor frecuencia de infecciones policlonales en la recurrencia que en el episodio inicial, mientras que los no gestantes mostraron mayor policlonalidad en el episodio primario que en la recurrencia. Esto se podría explicar porque las gestantes tienen mayor probabilidad de presentar recurrencias multiclonales debido a que no reciben la primaquina que actúa sobre los hipnozoítos.

La frecuencia de recaídas/reinfecciones detectada en las gestantes fue muy alta (41%, 17/41), incluso mayor que la reportada en otros estudios colombianos: 29% con seguimiento de 120 días ^33^ y 18% a 24% con 180 días de seguimiento ^28^^,^^47^. El uso de la qPCR además de la microscopía para la detección de la infección en este este estudio, puede explicar esa mayor frecuencia de recurrencias.

La recurrencias heterólogas fueron mucho más frecuentes que las homólogas, tanto en gestantes como en no gestantes, lo cual es consistente con el alto número de infecciones policlonales detectadas en ambas cohortes y coincide con otros estudios que reportan que las recurrencias heterólogas son generalmente policlonales y ocurren en la mayoría de zonas endémicas ^40^^,^^48^. Estos hallazgos también concuerdan con otro estudio colombiano que incluyó pacientes con *P. vivax* residentes en Turbo (municipio endémico) y Medellín (municipio no endémico) y reportó que los aislamientos de las infecciones recurrentes eran genéticamente diferentes a los aislamientos de la infección inicial ^29^. Pero contrastan con otro trabajo llevado a cabo en Turbo que reportó que 93% de las infecciones recurrentes por *P. vivax* era homólogas ^28^.

Hasta donde conocemos, este es el primer trabajo que explora la asociación entre el perfil inmune del hospedero con el genotipo parasitario en los episodios primario y recurrente, tanto en gestantes como en no gestantes de Colombia. No se encontraron diferencias estadísticamente significativas en el nivel de expresión de los mediadores inmunes entre infección inicial y recurrente, ni entre recurrencias homólogas y heterólogas. Por tanto, no hubo asociación del perfil inmune del hospedero con los genotipos plasmodiales en la infección inicial y recurrente; pero, quizás, esto se debe al escaso tamaño muestral disponible para el análisis, pues hay estudios que informan la existencia de diferencias entre los niveles de mediadores inmunes en la recurrencia, según sea, genéticamente homóloga o heteróloga al episodio inicial ^21^^,^^22^.

Como fortaleza se resalta la aproximación multifacética al problema en las gestantes, teniendo como grupo de comparación una cohorte de no gestantes. De igual manera, se aceptan como limitaciones el tamaño de muestra relativamente pequeño, la baja cantidad de marcadores moleculares usados, y el desconocimiento del estado nutricional de los participantes y de su estado de infección con otros parásitos como los intestinales, que son condiciones que pueden modular la respuesta inmune. Sin embargo, estas limitaciones no invalidan los hallazgos del estudio, pues las comparaciones se hacen entre pacientes de la misma región, que están expuestos a condiciones socioeconómicas similares, en general, precarias como en casi todas las zonas endémicas de malaria del mundo, por lo que se espera que su estado nutricional y de infección con parásitos intestinales también sea similar.

En conclusión, las gestantes están expuestas a alta frecuencia de recurrencias con alta variación genética, lo cual puede afectar el desarrollo de inmunidad protectora efectiva y generar desenlaces materno-neonatales adversos. Esto plantea la necesidad, no solo de fortalecer las acciones de prevención de la malaria por *P. vivax* en la gestante, sino también de optimizar el tratamiento antimalárico y encontrar alternativas al esquema hipnozoitocida disponible actualmente en casi todas las zonas endémicas de malaria.
